# Supplementary figures and images for: Genome-Wide Identification and Characterization of Caffeic Acid O-Methyltransferase Gene Family in Soybean
Source: Plants (Basel). 2021 Dec 20;10(12):2816. doi: 10.3390/plants10122816 (PMC8703356; doi:10.3390/plants10122816)

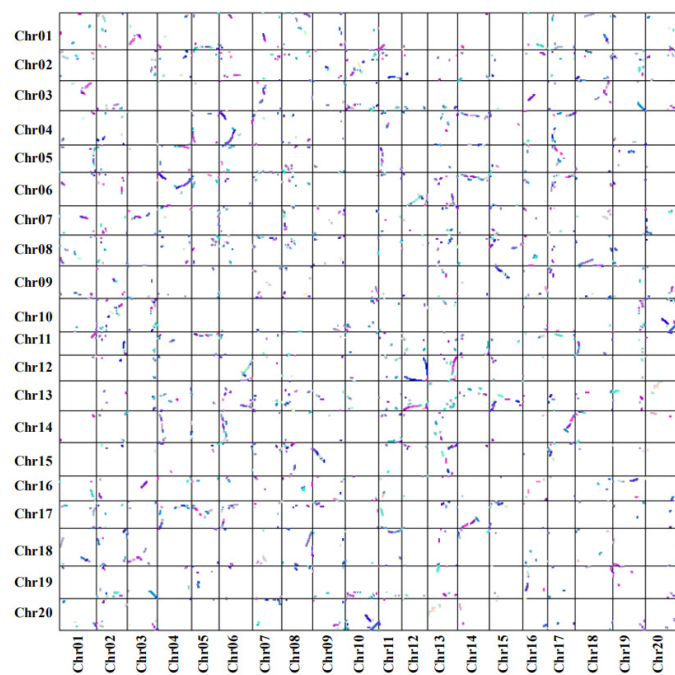

**Figure S1. Genomic collinearity analysis in soybean.**

Supplement: Supplementary file 1 [file plants-10-02816-s001.zip › Figure S1. Genomic collinearity analysis in soybean.pdf]
